# Effectiveness of auriculotherapy for anxiety, stress or burnout in health professionals: a network meta-analysis

**DOI:** 10.1590/1518-8345.6219.3708

**Published:** 2022-10-17

**Authors:** Oclaris Lopes Munhoz, Bruna Xavier Morais, Wendel Mombaque dos Santos, Cristiane Cardoso de Paula, Tânia Solange Bosi de Souza Magnago

**Affiliations:** 1 Universidade Federal de Santa Maria, Santa Maria, RS, Brazil.; 2 Hospital Alemão Oswaldo Cruz, São Paulo, SP, Brazil.; 3 Scholarship holder at the Coordenação de Aperfeiçoamento de Pessoal de Nível Superior (CAPES), Brazil.

**Keywords:** Auriculotherapy, Anxiety, Stress, Psychological, Burnout, Professional, Health Personnel, Network Meta-Analysis, Auriculoterapia, Ansiedade, Estresse Psicológico, Esgotamento Profissional, Pessoal da Saúde, Metanálise em Rede, Auriculoterapia, Ansiedad, Estrés Psicológico, Agotamiento Profesional, Personal de Salud, Metaanálisis en Red

## Abstract

**Objective:**

to analyze the effectiveness of auriculotherapy, when compared to the control group, placebo or usual treatment for anxiety, stress or burnout in health professionals.

**Method:**

a systematic review conducted in nine information sources, being selected experimental or quasi-experimental studies with auriculotherapy intervention in health professionals, compared to control, placebo or usual treatment groups. Descriptive analysis and network meta-analysis by means of direct and indirect comparison. Quality of the outcomes was assessed with the Confidence in Network Meta-analysis.

**Results:**

15 articles were included: 66.6% with Nursing teams and 53.3% with interventions involving semi-permanent needles. The *shen men*, brainstem, kidney, sympathetic, lung and liver acupoints predominated. There was a reduction in anxiety with semi-permanent needles (CI -8.18, -6.10), magnetic palettes (CI -7.76, -5.54), placebo (CI -5.47, -3.36) and seeds (CI -6.35, -4.05); as well as in stress with semi-permanent needles (CI -37.21, -10.88) and seeds with (CI -28.14, -11.70) and without a closed protocol (CI -36.42, -10.76). Meta-analysis was unfeasible for burnout; however, significant reductions were verified when it was treated with auriculotherapy.

**Conclusion:**

Auriculotherapy is effective to reduce anxiety and stress in health professionals; however, this assertion cannot be made in the case of burnout. It was evidenced that workers’ health is favored with the use of auriculotherapy.

## Resumo

**Objetivo:**

analisar a efetividade da auriculoterapia comparada ao grupo controle, placebo ou tratamento habitual para ansiedade, estresse ou *burnout* de profissionais da saúde.

**Método:**

revisão sistemática em nove fontes de informação, sendo selecionados estudos experimentais ou quase-experimentais com intervenção de auriculoterapia em profissionais da saúde, comparados com grupos controle, placebo ou tratamento habitual. Utilizou-se análise descritiva e metanálise em rede por comparação direta e indireta. A qualidade dos desfechos foi avaliada por meio do *Confidence in Network Meta-analysis*.

**Resultados:**

foram incluídos 15 artigos, sendo 66,6% com equipes de enfermagem, 53,3% com intervenção de agulhas semipermanentes. Predominaram os acupontos *shen men*, tronco cerebral, rim, simpático, pulmão e fígado. Houve redução de ansiedade com agulhas semipermanentes (IC -8,18, -6,10), paletas magnéticas (IC -7,76, -5,54), placebo (IC -5,47, -3,36) e sementes (IC -6,35, -4,05); de estresse com agulhas semipermanentes (IC -37.21, -10.88) e sementes com (IC -28.14, -11,70) e sem protocolo fechado (IC -36.42, -10.76). A metanálise foi inviável para *burnout*, mas foram constatadas reduções significativas quando tratado com auriculoterapia.

**Conclusão:**

a auriculoterapia é efetiva para reduzir ansiedade e estresse de profissionais da saúde, não sendo possível realizar esta afirmação para *burnout*. Evidenciou-se que a saúde do trabalhador é favorecida com uso da auriculoterapia.

## Resumen

**Objetivo:**

analizar la efectividad de la auriculoterapia, comparándola con el grupo control, el placebo o el tratamiento habitual para la ansiedad, el estrés o el *burnout* de los profesionales de la salud.

**Método:**

revisión sistemática en nueve fuentes de información, siendo seleccionados estudios experimentales o cuasiexperimentales con intervención de auriculoterapia en profesionales de la salud, comparada con grupos control, placebo o atención habitual. Análisis descriptivo y metaanálisis en red por comparación directa e indirecta. La calidad de los resultados se evaluó con *Confidence in Network Meta-analysis*.

**Resultados:**

Se incluyeron 15 artículos, 66,6% con equipos de enfermería, 53,3% con intervención de aguja semipermanente. Predominaron los puntos de acupuntura *shen men*, tronco encefálico, riñón, simpático, pulmón e hígado. Se observó una reducción de la ansiedad con agujas semipermanentes (IC -8,18, -6,10), paletas magnéticas (IC -7,76, -5,54), placebo (IC -5,47, -3,36) y semillas (IC -6,35, -4,05); del estrés con agujas semipermanentes (CI -37,21, -10,88) y semillas con (CI -28,14, -11,70) y sin protocolo cerrado (CI -36,42, -10,76). No fue posible realizar el metaanálisis para el *burnout*, pero se constató que hubo reducciones significativas cuando se trató con auriculoterapia.

**Conclusión:**

la auriculoterapia es eficaz para reducir la ansiedad y el estrés en los profesionales de la salud, pero no se puede afirmar que lo sea para el *burnout*. Se observó que la salud del trabajador mejora con el uso de la auriculoterapia.

**Highlights**

(1) Auriculotherapy is effective to reduce anxiety and stress in professionals.

(2) A significant reduction of burnout was verified when it was treated with auriculotherapy.

(3) Any auriculotherapy intervention is more effective than not performing it.

(4) Semi-permanent needles are more effective in relation to other materials.

## Introduction

Anxiety, stress and burnout are among the health problems that most affect the professionals working in health services^1^ ^-^ ^5^. The literature reveals that they are related, among other factors, to increased workload, physical and psychological illness, predisposition to cardiovascular diseases, and low immunity^1^ ^-^ ^4^.

The treatment for anxiety, stress or burnout is usually related to anxiolytic, calming and antidepressant medications and can be associated with psychological approaches, such as behavioral and interpersonal psychotherapies. Medications generate side effects, require adherence to their use and predispose individuals to relapses^2^ ^,^ ^4^ ^,^ ^6^. Thus, it is fundamental to use non-pharmacological intervention strategies aimed at minimizing the aforementioned problems and their consequences to the professionals’ health. In this context, auriculotherapy, as an Integrative and Complementary Practice (ICP), is effective for the treatment of physical, psychological and emotional disorders in health professionals^7^ ^-^ ^9^. 

Auriculotherapy is an acupuncture technique, which uses needles, microspheres or seeds to stimulate reflex points of the auricle that are directly related to the Central Nervous System, as well as to assist in the treatment of disorders of the human organism, favoring body homeostasis^9^ ^-^ ^10^. It is a safe practice, as it can be performed with non-invasive materials, causes minimal side effects, does not cause dependence and is easy to apply, which favors participation of the professionals^8^ ^-^ ^11^. There are two variants of auriculotherapy approach: the French one, which uses an auricular microsystem as reflexology of neurological action of the parasympathetic system, and the one based on Traditional Chinese Medicine (TCM), which is widespread and the most used^5^ ^,^ ^8^ ^-^ ^11^.

However, there is still variability related to the material used, the number of sessions and intervals between them, and the necessary follow-up period, which suggests the need to identify whether there are common characteristics of the intervention that can support a standardized treatment of the aforementioned diseases^7^ ^-^ ^9^ ^,^ ^12^ ^-^ ^13^. There are also no studies that jointly evaluate anxiety, stress and burnout. An umbrella review that sought diverse evidence on the efficacy of auriculotherapy as a therapeutic tool found that the practice has sufficient evidence to be used in the treatment of chronic pain and suggests evaluations of its effectiveness for other health conditions^14^. 

In addition, there are systematic review protocols registered to evaluate acupuncture as an adjuvant treatment for women with postpartum depression^15^ and for pain, physical function and quality of life in patients with rheumatoid arthritis^16^, also different from this proposal. Therefore, it is pertinent to search diverse scientific evidence about the topic in question, thus justifying conduction of this review.

This systematic review aimed to analyze the effectiveness of auriculotherapy, when compared to the control group, placebo or usual treatment for anxiety, stress or burnout in health professionals.

## Method

### Type of study

Systematic literature review, developed according to the JBI methodology for effectiveness studies^17^ and following the recommendations set forth in the *Preferred Reporting of Items to Include When Reporting a Systematic Review Involving a Network Meta-Analysis* (PRISMA NMA)^18^ for quality and transparency of writing, and the *Preferred Reporting Items for Systematic Review and Meta-Analyses* (PRISMA) for the flowchart corresponding to selection of the studies^19^. Review protocol registered in PROSPERO under code No.: CRD42020222009.

In order to formulate the review question, the PICO mnemonic^17^ was used, consisting in Population = Health care workers; Intervention = Auriculotherapy; Control (comparator) = Control Group, placebo or usual treatment; and Outcomes = Reduction of anxiety, stress or burnout. The review question was structured as follows: How effective is auriculotherapy in reducing anxiety, stress or burnout in health professionals, when comparing the intervention to the control group, placebo or usual treatment?

### Criteria to select the studies

The selection criteria of the original studies were as follows: experimental or quasi-experimental studies in English, Portuguese or Spanish, with a population of health workers (studies that included health professionals working in hospital care); auriculotherapy intervention (studies using the technique of pressure, acupuncture, or electrical stimulation, with the use of seeds, spheres, palettes, needles or micro needles); compared to control groups, placebo or usual treatment (absence of intervention, *sham* points or psychotropic treatment, monitoring with a psychologist/psychiatrist); with outcomes of anxiety, stress or burnout (positive or negative effects). There was no time restriction. 

### Sampling and definition of the sources of the primary studies

The search and selection process for the references took place in the following sources: MEDLINE, via PubMed, SCOPUS (*Elsevier*), EMBASE (*Elsevier*), *Literatura Latino-Americana e do Caribe em Ciências da Saúde* (LILACS) and *Modelos de Saúde e Medicamentos Tradicionais, Complementares e Integrativos nas Américas* (MOSAICO - VHL TCIM), by means of *Biblioteca Virtual da Saúde* (BVS), Web of Science (WoS), CINAHL, PSYCINFO and the Cochrane Library. Access was remote, via the Journals Portal of *Coordenação de Aperfeiçoamento de Pessoal de Nível Superior* (CAPES). The lists of references of the articles included were also verified.

### Search strategies in the information sources

Specific strategies were defined for each search source (Figure 1), which were applied on May 14^th^, 2021 The strategies were validated with the analysis made by a librarian from the Brazilian Center for Evidence-Based Health Care: JBI Excellence Center - JBI Brazil. The controlled descriptors were searched through the Descriptors in Health Sciences (*Descritores em Ciências da Saúde*, DeCS), MESH (Medical Subject Headings) terms and CINAHL headings, considering the particularities of each source; uncontrolled terms were also used. The strategies were combined with the “AND” and “OR” Boolean operators.

**Figure 1:** Strategies in the information sources of the systematic review. Santa Maria, RS, Brazil, 2022

Information sources	Strategy	References retrieved
MEDLINE (PubMed)	((((“auriculotherapy”[MeSH Terms]) OR (“acupuncture, ear”[MeSH Terms])) OR (“auriculotherapy”[All Fields])) OR (“acupuncture ear”[All Fields])) OR (“nada protocol”[All Fields]) AND (((((((((“anxiety”[MeSH Terms]) OR (“occupational stress”[MeSH Terms])) OR (“stress, physiological”[MeSH Terms])) OR (“burnout, psychological”[MeSH Terms])) OR (“anxiety”[All Fields])) OR (“occupational stress”[All Fields])) OR (“stress physiological”[All Fields])) OR (“burnout psychological”[All Fields])) OR (“stress”[All Fields])) OR (“burnout”[All Fields]) AND (((“health personnel”[MeSH Terms]) OR (“health personnel”[Title/Abstract])) OR (“workers”[Title/Abstract])) OR (“professionals”[Title/Abstract])	12
SCOPUS (Elsevier)	“auriculotherapy” OR “acupuncture, ear” OR “nada protocol” AND “anxiety” OR “occupational stress” OR “stress, physiological” OR “burnout, psychological” OR “stress” OR “burnout” AND “health personnel” OR “professionals” OR “workers” AND (LIMIT-TO (LANGUAGE, “English”) OR LIMIT-TO ( LANGUAGE , “Portuguese”) OR LIMIT-TO ( LANGUAGE, “Spanish”))	106
EMBASE (Elsevier)	‘auricular acupuncture’/exp OR ‘auricular acupuncture’ AND ‘anxiety’/exp OR ‘anxiety’ OR ‘job stress’/exp OR ‘job stress’ OR ‘mental stress’/exp OR ‘mental stress’ OR ‘professional burnout’/exp OR ‘professional burnout’ OR ‘burnout’/exp OR ‘burnout’	123
LILACS	(“auriculoterapia” OR “acupuntura auricular” OR “auriculotherapy” OR “acupuncture, ear” OR “nada protocol”) AND (“ansiedade” OR “estresse ocupacional” OR “estresse psicológico” OR “esgotamento psicológico” OR “estresse” OR “burnout” OR “anxiety” OR “occupational stress” OR “stress, physiological” OR “burnout, psychological” OR “stress” OR “ansiedad” OR “estrés laboral” OR “estrés psicológico” OR “agotamiento psicológico”) AND ( db:(“LILACS” OR “MTYCI”) AND la:(“pt” OR “es” OR “en”))	43
VHL TCIM	(“auriculoterapia” OR “acupuntura auricular” OR “auriculotherapy” OR “acupuncture, ear” OR “nada protocol”) AND (“ansiedade” OR “estresse ocupacional” OR “estresse psicológico” OR “esgotamento psicológico” OR “estresse” OR “burnout” OR “anxiety” OR “occupational stress” OR “stress, physiological” OR “burnout, psychological” OR “stress” OR “ansiedad” OR “estrés laboral” OR “estrés psicológico” OR “agotamiento psicológico”) AND ( db:(“LILACS” OR “MTYCI”) AND la:(“pt” OR “es” OR “en”))	13
Web of Science Core Collection (Clarivate analytics)	TS=(auriculotherapy OR “acupuncture ear” OR nada protocol) AND TS=(anxiety OR “occupational stress” OR “stress physiological” OR “burnout psychological” OR stress OR burnout) AND TS=(“health personnel” OR professionals OR workers) AND TI=(auriculotherapy OR “acupuncture ear” OR “nada protocol”) AND TI=(anxiety OR “occupational stress” OR “stress physiological” OR “burnout psychological” OR stress OR burnout) AND TI=(“health personnel” OR professionals OR workers) AND AB=(auriculotherapy OR “acupuncture ear” OR “nada protocol”) AND AB=(anxiety OR “occupational stress” OR “stress physiological” OR “burnout psychological” OR stress OR burnout) AND AB=(“health personnel” OR professionals OR workers) AND AK=(auriculotherapy OR “acupuncture ear” OR “nada protocol”) AND AK=(anxiety OR “occupational stress” OR “stress physiological” OR “burnout psychological” OR stress OR burnout) AND AK=(“health personnel” OR professionals OR workers) Índices=SCI-EXPANDED, SSCI, A&HCI, CPCI-S, CPCI-SSH, ESCI Tempo estipulado=Todos os anos	9
CINAHL (EBSCO)	TX ( auriculotherapy OR “acupuncture ear” OR “nada protocol” ) AND TX ( anxiety OR “occupational stress” OR “stress physiological” OR “burnout psychological” OR stress OR burnout ) AND TX ( “health personnel” OR professionals OR workers )	47
APA PsycINFO (EBSCO)	“auriculotherapy” OR Any Field: “acupuncture ear” OR Any Field: “nada protocol” AND Any Field: “anxiety” OR Any Field: “occupational stress” OR Any Field: “stress physiological” OR Any Field: “burnout psychological” OR Any Field: “stress” OR Any Field: “burnout” AND Any Field: “health personnel” OR Any Field: “professionals” OR Any Field: “workers”	6
Cochrane Central Register of Controlled Trials (CENTRAL)	“auriculotherapy” OR “acupuncture, ear” OR “nada protocol”	429

The articles accessed through the search strategies were imported into the *Mendeley* software program. Duplicates were merged, titles and abstracts were read, and the articles included were read in full in a double independent manner. There were no divergences for the stage corresponding to search and selection of the references in the consensus meeting.

### Data extraction

In order to extract the information, a form was prepared in *Excel* ^®^, where the following data were considered: identification of the article (authors, title, journal, year and language of publication), objectives and methodological property (type of study, sample, size of the groups, follow-up losses, research instruments and outcomes evaluated); sociodemographic and clinical characteristics; interventions performed (treatment line, use of closed protocol or systemic analysis, location technique and points applied, number of sessions and interval between them, material used for the therapy); main results (effects of improvement or worsening in the health conditions, effect size, statistical differences, side effects) and conclusions. The characteristics of the intervention were extracted and adapted according to the precepts set forth in the *Revised Standards for Reporting Interventions in Clinical Trials of Acupuncture* (STRICTA)^20^.

In order to minimize biases, extraction of the information included in the synthesis of the evidence was developed independently by two reviewers with experience in the theme of auriculotherapy. In this process, each reviewer extracted the data by means of an *Excel* ^®^ form by using the data validation tool. There were no divergences in the consensus meeting. 

### Critical evaluation of the studies selected

The evaluation of the methodological quality corresponding to the articles included was also developed in a double and independent manner. The divergences identified in the consensus meeting were solved by a third reviewer. The instruments used were those recommended by the JBI^17^ for randomized clinical trials (individual participants in parallel groups), quasi-experimental studies (non-randomized experimental studies) and multiple cases. The methodological quality level was determined as follows: reasonable quality = less than 40% of the items presented; moderate quality = between 41 and 80% of the items presented; good quality = more than 80% of the items presented^21^. All articles were considered for the synthesis of the evidence, without determining the cutoff point for inclusion, as recommended by the JBI^17^, and the result of the critical evaluation was presented for each study.

### Synthesis of the diverse evidence found

A network meta-analysis was performed by direct and indirect comparison of random effects model, through the *Network Meta-Analysis* (NMA), combining estimates of different interventions in a single analysis. Random-effects models are appropriate when the number of studies is large enough, i.e., sufficient to support generalization inferences beyond the studies included. The recommendation is to use the fixed-effects model when the number of studies included is lower than five. Through this analysis it is possible to make indirect comparisons between pairs of interventions not evaluated in clinical research studies. This comparison also allows estimating the relative classification of an intervention in relation to an outcome of interest^18^ ^,^ ^22^ ^-^ ^23^.

Statistical heterogeneity was considered to perform this analysis. For the case of considering the fixed-effects model, it is assumed that all studies included in a meta-analysis are estimating a single true effect size, but if there is statistical heterogeneity, such model is not adequate. On the other hand, the random-effects model must be considered when it cannot be assumed that there is true homogeneity^22^.

The criterion of likelihood of a size effect in common was also considered. In fixed-effects models, it is assumed that there is a size effect in common. In random models, each study estimates a different underlying actual effect, and these effects have a distribution. Consequently, in the current report, the respective criteria presented were considered and the analysis was performed considering random effects^22^ ^-^ ^23^.

Transitivity was considered to perform the analysis, as indirect comparisons can suffer biases from the studies included. Transitivity requires intervention A to be similar when it appears in studies A versus B and A versus C for characteristics (effect modifiers) that may affect both relative effects. Transitivity requires imagining the interventions being simultaneously compared in a single multiple-branch randomized trial^18^ ^,^ ^22^ ^-^ ^23^. 

The Webapp Confidence in Network Meta-Analysis (CINeMA) software was used to perform the network meta-analysis and to evaluate the overall quality of the evidence. It includes a *netmeta* package of the R® software to analyze relative effects and heterogeneity of research studies^22^ ^-^ ^23^. Evaluating the methodological quality follows a structure that considers six domains: bias within the study, reporting bias, indirect bias, imprecision, heterogeneity and inconsistency. Finally, judgments in the domains are summarized in a single confidence rating of “high,” “moderate,” “low,” or “very low”^22^ ^-^ ^23^.

## Results

**Figure 2:**
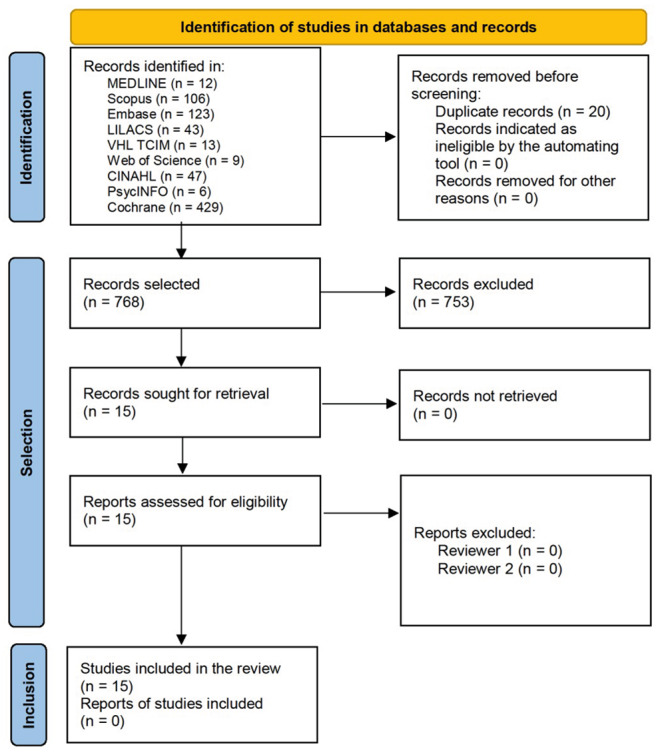
Flowchart corresponding to selection of the studies adapted from the Preferred Reporting Items for Systematic Review and Meta-Analyses (PRISMA)^19^. Santa Maria, RS, Brazil, 2022

From the search strategies in the information sources, it was possible to identify 788 references, of which 20 were duplicates and were considered only once. Thus, 768 productions were read in the phase of selection by titles and abstracts. Of these, 753 were excluded for not meeting the selection criteria. In the following stage, 15 articles^8^ ^-^ ^9^ ^,^ ^12^ ^-^ ^13^ ^,^ ^24^ ^-^ ^34^ were listed for full-reading and remained for the synthesis of the evidence (Figure 2). It is noted that four articles included did not contain all the information listed for extraction and, on this occasion, contact was made with the authors via email; one of them^32^ provided the requested results, two^13^ ^,^ ^24^ reported that it would not be possible to do so, and another one^33^ did not answer (after two attempts). Thus, the three articles^13^ ^,^ ^24^ ^,^ ^33^ with missing data for the NMA were presented in the narrative synthesis, as recommended by the JBI^17^.

Of the 15 articles included, the publications occurred between 2009 and 2021^8^ ^-^ ^9^ ^,^ ^12^ ^-^ ^13^ ^,^ ^24^ ^-^ ^34^, with 11 (73.3%)^8^ ^,^ ^12^ ^,^ ^24^ ^-^ ^27^ ^,^ ^29^ ^-^ ^32^ ^,^ ^34^ research studies developed in Brazil, three (20.0%)^9^ ^,^ ^13^ ^,^ ^33^ in the United States of America (USA) and one (6.7%) in Italy^28^. In addition, 10 (66.6%)^8^ ^,^ ^12^ ^,^ ^25^ ^-^ ^31^ ^,^ ^33^ are Randomized Clinical Trials (RCTs), four (26.7%)^9^ ^,^ ^13^ ^,^ ^24^ ^,^ ^32^ are quasi-experimental studies of the before-and-after type, and one (6.7%)^34^ is a multiple case study. Auriculotherapy was applied to 860 participants (sum of all samples of the articles); 10 (66.6%) studies included the Nursing team^(8,24-27.29-32,34)^, four (26.7%) all categories of health professionals^9^ ^,^ ^14^ ^,^ ^28^ ^,^ ^33^ and one (6.7%), only nurses^12^. Figure 3 chronologically synthesizes the other characteristics of the articles.

**Figure 3:** Characteristics included for the synthesis of the systematic review articles, authorship, journal, year of publication, outcomes evaluated, participants, number and interval between auriculotherapy sessions, monitoring follow-up, ear points, intervention materials used and main results. Santa Maria, RS, Brazil, 2022

Authorship of the study, journal and year	Outcomes evaluated	Participants, number and interval between the auriculotherapy sessions and follow-up	Ear points	Material used for the auriculotherapy sessions	Main results
Giaponesi ALL, et al. Rev. Nursing, 2009.^24^	- Stress	- 41 Nursing professionals - 8 sessions, with 7-day intervals - No follow-up	- *Shen men* and brainstem	- This information is not included in the article	The mean for stress (27%) was reduced to 10%, as 17% of the subjects started to show little stress and 35 individuals (85.4%) reported an improvement in their Stress level.
Kurebayashi LFS, et al. Rev. *Acta Paulista de Enf*, 2012.^25^	- Stress	- 49 Nursing professionals - 8 sessions, with 7-day intervals - Follow-up = 15 days	- *Shen men*, kidney and brainstem	- Semi-permanent needles	There was a significant difference between the Control Group and Group 3 (more experienced therapists) (p=0.036), between LSS^*^3/LSS^*^1 and between LSS^*^4/LSS^*^1 during follow-up (p=0.033). Group 2 (less experienced) showed significance for LSS^*^4/LSS^*^1 (p=0.059).
Kurebayashi LFS, et al. *Rev Esc Enferm USP*, 2012.^26^ Kurebayashi LFS, et al. *Rev. Latino-Am. Nursing*, 2012.^27^ Kurebayashi LFS, et al. *Rev. Eletr Enf*, 2014.^29^	- Stress	- 75 Nursing professionals - 8 sessions, with 7-day intervals - Follow-up = 15 days	- *Shen men*, kidney and brainstem	- Semi-permanent needles - Mustard seeds	Statistically significant differences were verified when all three groups were compared, at 4 different moments (LSS^*^1, LSS^*^2, LSS^*^3, LSS^*^4). The significance level between the differences of the results was p=0.020 between LSS^*^3 and LSS^*^1 and p=0.003 when LSS^*^4 and LSS^*^1 were compared. The seed group did not present statistically significant differences.
Reilly PM, et al. Rev Dimen of Crit Care Nursing, 2014.^13^	- Anxiety - Burnout	- 37 health professionals - 5 sessions, with 7-day intervals - No follow-up	- *Shen men*, sympathetic, lung, liver and kidney	- Stainless steel ear acupuncture needles	There was a significant reduction in state anxiety (p=0.000) and trait anxiety (p=0.007), and for burnout (p=0.006) in the treated participants when compared to the baseline.
Gagliardi G, et al. Rev. Medical Acupuncture, 2014.^28^	- Anxiety	- 20 health professionals - 2 sessions with 2-week intervals - No follow-up	- Points according to the individual evaluation of each participant (without a closed protocol)	- Real needles - Fake needles	There was a significantly higher reduction of anxiety in the real needle group for the NRS^†^ anxiety (p<0.01) and state anxiety (p<0.005) scores.
Kurebayashi LFS, et al. Rev. Latino-Am. Nursing, 2014.^30^ Kurebayashi LFS, et al. *Rev. Bras Enferm*, 2015.^31^	- Stress	- 175 Nursing professionals - 12 sessions, with 7-day intervals - Follow-up = 30 days	*- Shen men*, brain tem, kidney and liver *yang* 1 and 2	- Semi-permanent needles	Stress was reduced (p<0.05) in both intervention groups. There were significant differences between the stress mean values in the second evaluation after 12 appointments (LSS^*^2) (F=21.92/p=0.000) an in the 30-day follow-up (F=7.59/0=0.001); as well as in the second evaluation between the Control and Intervention groups (p=0.000). In the third evaluation (LSS3), the differences were between the Control group and protocol (p=0.004) and between the Control group and no protocol (p=0.002).
Kurebayashi LFS, et al. Rev. Latino-Am. Nursing, 2017.^8^	- Anxiety	- 133 Nursing professionals - 10 sessions, with 3-day intervals - No follow-up	- *Shen men*, tranquilizer, thalamus, sympathetic, zero point	- Semi-permanent needles - Mustard seeds	There were significant differences for the reduction of anxiety after 10 sessions. The group with semi-permanent needles reached a large effect and 17% reduction.
Buchanan TM, et al. Rev. Dimensions of Critical Care Nursing, 2018.^9^	- Anxiety	- 42 health professionals - 5 sessions, with 7-day intervals - No follow-up	- *Shen men*, sympathetic, lung, liver and kidney	- Disposable needles	Seven percentiles of state and trait anxiety decreased by approximately 15 points. When comparing the post-intervention scores to the baseline, there is a significant reduction in state anxiety (from 37.64 to 32.6; p=0.0001) and trait anxiety (from 38.14 to 34.64; p=0.0001).
Araújo JS, et al. *Rev. Enferm UFPE*, 2018.^32^	- Stress	- 16 Nursing professionals - 8 sessions, with 7-day intervals - No follow-up	- *Shen men* and brainstem	- Crystals	The nurses did not have their stress level reduced. At baseline there were five participants (83.3%) in the stress-free phase and one (16.6%) in resistance; at the 8^th^ application, all nurses were stress-free; and there was a significant reduction in stress (p=0.317). During follow-up, one participant returned to the resistance phase. There was stress reduction in nursing assistants between baseline and follow-up (p=0.034).
Prado JM, et al. *Rev Esc Enferm USP*, 2018.^12^	- Stress	- 168 nurses - 12 sessions, with 3-day intervals - Follow-up = 15 days	- *Shen men* and brainstem (intervention) - External ear and cheek area (placebo)	- The material used is not specified	There were statistical differences in the analysis between all three groups (p=0.000). In the *post hoc* period there was a difference for the auriculotherapy group between the baseline and the 2^nd^ evaluation, after eight sessions, remaining in the third evaluation (12 sessions) until follow-up (p=0.000).
Olshan-Perlmutter M, et al. Rev. Applied Nursing Research, 2019.^33^	- Anxiety - Burnout	- 98 health professionals - 6 sessions, with 7-day intervals - No follow-up	- *Shen men*	- Magnetic palettes	Group 1 (treatment during the first six weeks) and Group 2 (started treatment at week 7) significantly improved (p<0.05) the anxiety levels after the treatment. When compared to the baseline there was also a significant improvement for burnout in each participant (p<0.05).
Oliveira CMC, et al. *Rev. Eletr. Enf*, 2021.^34^	- Anxiety - Stress	- 41 Nursing professionals - 1 session - Follow-up = 15 days	- *Shen men*, kidney, sympathetic, joy, anxiety, antidepressant, heart, endocrine, lung and muscle relaxation	- Mustard seeds	The anxiety levels were significantly reduced: medians from six to four (p<0.001). The stress mean values were significantly reduced from 19.37 to 11.95 (p<0.001).

*List of Stress Signs and Symptoms; ^†^Numerical Rating Scale for Anxiety

There was predominance of Traditional Chinese Medicine (N=15; 100%) when compared to the French approach, with eight studies (53.3%)^8^ ^,^ ^13^ ^,^ ^25^ ^-^ ^27^ ^,^ ^29^ ^-^ ^31^ applying semi-permanent needles. As for the location technique, the use of a point locator (53.3%)^8^ ^,^ ^25^ ^-^ ^27^ ^,^ ^29^ ^-^ ^32^ predominated; six did not clarify the technique used (40.0%)^9^ ^,^ ^12^ ^-^ ^13^ ^,^ ^24^ ^,^ ^33^ ^-^ ^34^ and one (6.7%) made use of an algometer with 250 grams of maximum pressure^28^.

Figure 4 shows the characteristics of the studies regarding the instruments for measuring the outcomes, intervention and/or placebo groups, mean values (median) and standard deviation (interquartile range) at the beginning and end (pre- and post-interventions).

**Figure 4:** Characteristics of the studies regarding the instruments for measuring the outcomes, intervention and/or placebo groups, mean values (median) and standard deviation (interquartile range) at the beginning and end (pre- and post-interventions). Santa Maria, RS, Brazil, 2022

Outcome	Study	Measuring instruments	Intervention Group				**Control Group/Placebo/*Sham***			
			Group	Mean (I=Initial; F=Final)	Standard Deviation (I=Initial; F=Final)	Pop^*^	Group	Mean (I=Initial; F=Final)	Standard Deviation (I=Initial; F=Final)	Pop^*^
Anxiety	Gagliardi G, et al.^28^	STAI^†^ NRS^‡^	Real needle	STAI^†^ I: 45.50 F: 41.00	STAI^†^ I: 3.38 F: 3.15	20	Fake needle	STAI^†^ I: 45.10 F: 43.60	STAI^†^ I: 3.8 F: 2.01	20
				NRS^‡^ I: 3.1 F: 1.7	NRS^‡^ I: 0.76 F: 0.75			NRS^‡^ I: 3.0 F: 2.3	NRS^‡^ I: 0.73 F: 0.86	
	Reilly PM, et al.^13^	STAI^†^	Single group (needle)	Trait I: 37.20 F: 34.20	Trait I: 7.70 F: -^††^	37	Does not apply			
				State I: 38.30 F: 32.30	State I: 7.70 F: -^††^					
	Kurebayashi LFS, et al.^8^	STAI^†^	Seeds	I: 49.30 F: 42.80	I: 7.90 F: 10.50	35	No intervention	I: 48.0 F: 46.7	I: 9.3 F: 10.4	31
			Needle	I: 51.60 F: 42.90	I: 9.80 F: 6.30	34	Adhesive tape	I: 49.50 F: 44.10	I: 8.70 F: 8.90	33
	Buchanan TM, et al.^32^	STAI^†^	Single group (needle)	Trait I: 38.14 F: 34.62	Trait I: 9.28 F: 8.74	42	Does not apply			
				State I: 37.64 F: 32.60	State I: 9.44 F: 9.34					
	Olshan-Perlmutter M, et al.^33^	GAD-7^§^	Initial treatment (Magnetic palettes)	I: 6.14 F: 3.65	I: 4.80 F: -^††^	51	Waiting list	I: 5.91 F: 5.65	I: 4.9 F: -^††^	47
	Oliveira CMC, et al.^34^	DASS-21^||^	Single group (seeds)	I: 6 (median) F: 4 (median)	I: 4-16 (IQR^‡‡^) F: 0-7 (IQR^‡‡^)	41	Does not apply			
Stress	Kurebayashi LFS, et al.^27^ Kurebayashi LFS, et al.^29^	LSS^¶^	Needles	I: 66.82 F: 48.48	I: 18.56 F: 27.35	27	No intervention	I: 54.36 F: 55.77	I: 15.90 F: 30.98	22
			Seeds	I: 63.27 F: 53.36	I: 26.05 F: 32.72	26				
	Kurebayashi LFS, et al.^30^ Kurebayashi LFS, et al.^31^	LSS^¶^	With protocol (needles)	I: 62.26 F: 48.50	I: 21.50 F: 22.90	58	No intervention	I: 57.76 F: 63.21	I: 17.64 F: 26.85	58
			No protocol (needles)	I: 65.00 F: 47.22	I: 22.62 F: 23.87	59				
	Prado JM, et al.^12^	LSS^¶^	Points indicated for stress	I: 72.40 F: 41.30	I: 17.90 F: 16.40	43	No intervention	I: 69.30 F: 66.80	I: 17.80 F: 27.60	43
							*Sham* points	I: 66.70 F: 51.80	I: 17.30 F: 27.00	47
	Oliveira CMC, et al.^34^	DASS-21^||^	Single group (seeds)	I: 19.37 F: 11.95	I: 10.61 F: 8.51	41	Does not apply			
Burnout	Olshan-Perlmutter M, et al.^33^	PQOL^**^	Initial treatment (Magnetic palettes)	I: 22.30 F: 20.42	I: 5.11 F: -^††^	51	Waiting list	I: 20.08 F: 21.56	I: 5.25 F: -^††^	47
	Reilly PM, et al.^13^	PQOL^**^	Single group (needle)	I: 22.90 F: 21.30	I: 5.40 F: 6.00	37	Does not apply			

*Population; ^†^State-Trait Anxiety Inventory; ^‡^Numerical Rating Scale for Anxiety; ^§^Generalized Anxiety Disorder; ^||^DASS-21- Depression, Anxiety, and Stress Scale-21; ^¶^List of Stress Signs and Symptoms; ^**^Professional Quality of Life Scale; ^††^Missing information in the article; ^‡‡^Interquartile Range

Regarding the side effects reported in the articles included in this review, there were cases of nightmares^24^ ^-^ ^26^ ^,^ ^28^, one case of itching^33^ and another of pain^8^. A number of research studies^30^ ^-^ ^31^ report occurrence of side effects in three cases, although not specifying which. One study^34^ highlights that the participants had no side effects and this information was not presented in others^9^ ^,^ ^12^ ^-^ ^13^ ^,^ ^24^ ^,^ ^28^ ^,^ ^32^.

In the critical evaluation^17^ of the methodological quality of the articles included, four of the RCTs^26^ ^,^ ^28^ ^,^ ^31^ ^,^ ^33^ showed reasonable quality and six^8^ ^,^ ^12^ ^,^ ^25^ ^,^ ^27^ ^,^ ^29^ ^-^ ^30^ moderate quality; none of them reached good quality. All the quasi-experimental^9^ ^,^ ^13^ ^,^ ^24^ ^,^ ^32^ and multiple-case^34^ studies were of moderate quality. 

Of the items of the instruments used^17^, it is noted that the questions number 5 (*Were those who administered the treatment blinded to treatment allocation?*), 6 (*Were the outcome evaluators blinded to treatment allocation?*) and 11 (*Were the results measured in a reliable way?*) were not included in any RCT of the articles included; and questions number 10 (*Were the results measured in the same way for the treatment groups?*) and 13 (*Was the study design appropriate and were any deviations taken into account in conduction and analysis of the study?*) were the most covered, with percentages of 90% and 100%, respectively. 

The reports of the quasi-experimental studies included questions 1 (*Is it clear in the study what the cause and the effect are?*), 2 (*Were the participants included in any similar comparison?*), 5 (*Were there several measurements of the outcome before and after the intervention/exposure?*) and 7 (*Were the results of the participants included in any comparison measured in the same way?*) (100.0%); on the other hand, questions number 3 *(Were the participants included in any comparisons that received similar treatment/care, in addition to the exposure/intervention of interest?*), 4 (*Was there a control group?*) and 6 (*Was follow-up complete and, if not, were differences between groups in terms of follow-up adequately described and analyzed?*), did not (0.0%). 

Table 1 presents the analysis comparing the different groups and materials of the intervention by outcome.

**Table 1:** Network meta-analysis of the direct and indirect comparisons of results about the effectiveness of auriculotherapy for anxiety and stress in different groups. Santa Maria, RS, Brazil, 2022

ANXIETY				
Semi-permanent needles	-0.49 (-1.12, 0.14)	**-2.72 (-3.25, -2.20)**	**-1.92 (-2.86, -1.02)**	**-7.14 (-8.18, -6.10)**
	Magnetic palettes	**-2.23 (-2.58, -1.88)**	**-1.45 (-2.45, -0.45)**	**-6.65 (-7.76, -5.54)**
		Placebo	0.78 (-0.15, 1.72)	**-4.42 (-5.47, -3.36)**
			Seeds	**-5.20 (-6.35, -4.05)**
				No intervention
STRESS				
Semi-permanent needles	-16.01 (-33.47, 1.46)	-4.12 (-17.29, 9.05)	-0.46 (-17.90, 16,984)	**-24.05 (-37.21, -10.88)**
	Placebo	11.88 (-0.97, 24.74)	15.55 (-1.66, 32.76)	-8.04 (-20.91, 4.83)
		Seeds	3,663 (-9.16, 16.49)	**-19.92 (-28.14, -11.70)**
			Semi-permanent needle (without a closed protocol)	**-23.59 (-36.42, -10.76)**
				No intervention

Note: Placebo and no intervention act as common comparators. The comparisons between the interventions must be read from left to right. For peer meta-analysis (top right), a WMD (absolute difference between means) above 0 favors the line definition treatment. For the comparisons in the opposite direction, the negative values must be converted into positive ones, and vice-versa. The significant results are indicated in bold type.

The general evaluation of the quality of the evidence by CINeMA showed that the evidence was low for anxiety and stress, considering all treatments evaluated (semi-permanent needles, magnetic palettes, placebo, seeds or without intervention). It is also noted that it was not possible to perform this type of meta-analysis for burnout for not having at least two studies that measure this outcome with the same methodological design.

## Discussion

The diverse evidence of this systematic review reinforce the benefits of auriculotherapy for the reduction of anxiety and stress in health professionals, revealing different forms of intervention and evaluation of the outcomes. Nevertheless, although heterogeneity was found among the studies included, it was possible to obtain a synthesis of the best scientific evidence on the theme, which contributes to the notoriety and use of auriculotherapy in the clinical practice.

The productions revealed predominance of participation of the Nursing team as a population, as well as the fact that those responsible for applying the intervention were auriculotherapist nurses, a scenario that is line with the study that evaluated the technique for anxiety, stress and depression in adults and older adults^11^. Regarding this panorama, in the first place, it is known that these diseases are prevalent in Nursing teams, as this is the category with the highest number of workers in health institutions; in addition to that, they are the ones who most adhere to participation in research studies on the theme^2^ ^,^ ^4^ ^-^ ^5^.

Added to that, the ear acupuncture practice is recognized as a nurse’s specialty, with legal support for application of the technique^35^ , which can be practiced by other professional categories. As well as other integrative practices, auriculotherapy is recognized as a Nursing intervention with a specific language^36^ ^-^ ^37^, corroborating for care with scientific basis. 

With regard to the auriculotherapy technique, while there was variability in the applications, most of the studies used needles for the interventions, with a mean of eight sessions and no follow-up. Systematic reviews that evaluated the effectiveness of auriculotherapy for the treatments for chronic spinal pain in adults^38^ and for obesity^39^ also verified that there is no specific protocol for choosing ear points. On the one hand, this scenario is in line with the assumptions of TCM^40^ ^-^ ^41^, which emphasize the importance of an individual approach to the relief of body disorders; however, it is encouraged that closed treatment protocols, with specific points, be tested in clinical research studies with a view to standardizing interventions according to health problems. Regarding the predominance for using semi-permanent needles, in addition to the literature revealing better benefits of this material, they maintain the active points continuously and do not require stimulation by the individual^8^ ^,^ ^38^.

As for the comparison groups for the interventions tested, it was verified that the control group (without any intervention or with *sham* points - not indicated for the outcome of interest) is the most used in research studies in the area. This perspective corroborates findings related to the use of auriculotherapy for the treatment of chronic spinal pain^38^, chronic renal failure^42^, cancer patients^43^ and for the reduction of Body Mass Index in overweight/obese patients^44^. In this context, it is necessary to reflect on the aspect referring to blinding of the participants, given that auriculotherapy is a visible intervention and that, in theory, only the therapist who applies it knows who is allocated to each group. However, many participants share time and space every day and can perceive differences between each other, mainly when they are not subjected to any intervention. Therefore, comparisons made with *sham* points minimize the aforementioned bias, considering that all the participants subjected to treatments undergo auriculotherapy.

In addition to comparison criteria with intervention groups, it is worth mentioning the diverse evidence on placebo/*sham* groups regarding effectiveness in stress relief, as statistically significant reductions were found^12^ ^,^ ^31^. In ear acupuncture, the effect of placebo can be related to emotional and psychological aspects, considering that the participants understand that they can receive/are receiving a treatment, in addition to experiencing positive expectations and the probable creation of a bond from the multiple sessions^12^ ^,^ ^31^. Such factors can be useful as strategies to cope with the stressors.

The *shen men*, brainstem, kidney, sympathetic, lung and liver ear points were the most used for the treatment of the outcomes evaluated. It is noted that they are used for the following: the first, control of the excitatory and inhibitory action of the cerebral cortex, with tranquilizing, analgesic and anti-allergic effect; the second, brain disorders, also with sedation function; the third favors health preservation, benefiting brain function; the sympathetic point is indicated for circulatory and neurovegetative changes; and the lung and liver points act on sadness and anger, respectively^41^. Such points are close to the NADA (*National Acupuncture Detoxification* Association) protocol, which is widely used and disseminated in the auriculotherapy area^41^. 

Therefore, as for the technique used to locate points, the use of a point finder of the ear type was predominant. With this technique it is possible to identify the most sensitive points in the individual and verify the presence of changes that had not been previously noticed. In addition to that, it is possible to verify regions or points where pain is more intense. Therefore, the diagnosis of the reagent point is always the one with the highest intensity. However, pain varies according to each patient, which reasserts the need for a comprehensive evaluation, especially with the reactions during the intervention^41^. In general, the ideal is that the pain reagent point also presents edema (locker) and is still related to the patient’s complaint (clinical).

Using the electrodiagnostic method to locate acupoints is also a possibility. Its difference when compared to the feelers is that this detection takes place by means of an electrical response. Thus, the electrodiagnosis is made by means of a device with sensitivity control, point indicator light and sound emitter. Some models have a kind of “stick” that is used for electrical grounding^41^. In addition, this technique has been recommended for the development of clinical research studies with auriculotherapy, considering the possibility of better precision and standardization in the detection of ear points. 

Regarding the side effects related to applying auriculotherapy, it was verified that they are scarcely frequent, as there were reports of nightmares, itching and pain. In the nightmare cases, according to TCM, they reflect reactions that are not directly related to the therapy^41^. Pain is a local event and is among the most reported, a scenario also evidenced in other systematic analyses^11^ ^,^ ^14^ ^,^ ^43^; it also occurs regardless of the material used^41^. It is noted that the aforementioned reactions are momentary and bearable, as well as they tend to decrease after the second application day. 

From the critical evaluation of the methodological quality^17^ of the articles included in this review, it was verified that, in general, they are of reasonable and moderate quality. This scenario is close to other systematic reviews about auriculotherapy in which weaknesses were also identified^11^ ^,^ ^14^, which exerts impacts on the methodological rigor of research on this theme. Given the above, it is understood that the use of instruments for planning, development and subsequent writing of intervention reports constitutes an important strategy to minimize biases.

In this scenario, the aspects not covered by the RCTs are those related to blinding of the therapist and of the outcome evaluators, as well as to the fact that the results are measured in a reliable way^17^. To such end, auriculotherapy requires that the person in charge of the intervention is aware of the ear points during application; therefore, this individual cannot be blinded. However, it is recommended that, in clinical research studies, the evaluators responsible for measuring the outcomes are not aware of which participant is in each group; otherwise, there is a risk of distorting the results^45^. Furthermore, it was verified that there are no details about measuring reliability, whether it took place equally for all participants; in addition to how many evaluators there were and if they were trained. These requirements need to be contemplated and, if not, their non-inclusion must be justified and described in the research report^17^ ^,^ ^45^. 

As for the quasi-experimental studies, none contemplated/described aspects related to whether or not there were comparisons with the treatment of interest, if there was a control group, or if the follow-up was complete or not^17^. There needs to be clarity regarding the differences between groups in terms of treatments or care received, that is, if during the intervention of interest there were other exposures; if so, its effect is compromised^41^. The incentive to compare the intervention with a Control Group is related to strengthening validity of the inferences. In addition, related to the monitoring, follow-up losses, their justifications and how they were analyzed should be described, given that internal validity of a study can be threatened when there are important differences between compared groups^17^ ^,^ ^45^. Such aspects must also be described in detail^45^. 

Drawing an overview directed to the effectiveness of auriculotherapy, through the network meta-analysis, it was evidenced that performing any type of intervention with auriculotherapy, including placebo (*sham* points), is more effective to reduce anxiety and stress in health professionals than not intervening. Therefore, intervening with auriculotherapy is more effective to reduce anxiety and stress when compared to the control group, that is, it is an advantageous practice to alleviate these diseases. 

Given the above, it becomes necessary to discuss about the relationship between the neuropsychobiology of anxiety, stress and burnout and the effectiveness evidenced. To this end, such problems are close to each other with regard to signs and symptoms, which are predominantly the following: agitation, nervousness, insomnia, sadness, fear of the unknown, fatigue, tension, discouragement and mental exhaustion, among others^1^ ^-^ ^4^. These responses are coordinated reactions that occur due to aversive stimuli, preparing the organism for fight or flight, ways of coping that activate the autonomic nervous system and result in cortisol release by the adrenal glands^46^ ^-^ ^47^. 

In this sense, it is noted that this response is regulated by the Hypothalamus-Hypophysis-Adrenal (HPA) axis. Thus, cortisol is released by the adrenal gland in response to an increase in the adrenocorticotropic hormone blood levels. Hypothalamic neurons also secrete corticotrophin and are regulated by the amygdala and hippocampus. Therefore, when inappropriate activation occurs (recurrent exposure to stressors), there are high levels of circulating cortisol, which is related to the disorders herein discussed. Continuous exposure to cortisol predisposes to neuronal death and to failures in the ability to perform routine and memory functions^46^ ^-^ ^47^. Thus, when stimulated, the acupoints chosen for the treatment of anxiety, stress or burnout found in this review provide reflex connections with other parts of the body, through neural pathways, resulting in viscero-somatic reflexes that seek homeostatic action on the HPA axis, responsible for cortisol release^30^ ^,^ ^41^. 

It was also verified that semi-permanent needles are more effective when compared to other materials. This fact corroborates findings from another study^38^. The justification for this difference is related to two issues: first, spherical stimulators are “patient-dependent”, as their effectiveness is directly related to the stimulus with direct pressure that the patient must perform at least three times a day, although they are less invasive, safer and with less risk of ear injury; second, the needles, widely disseminated, when applied, cause pain and local inflammation, causing the point to remain active, without the need for manual stimulation, although they can cause greater discomfort^38^ ^,^ ^41^. 

In relation to burnout, it was not possible to perform the meta-analysis because two studies that do not share the same methodological design were found. These studies verified statistically significant reductions in its prevalence when compared to baseline, in health professionals exposed to treatment with needles^28^ and magnetic palettes^33^. The burnout syndrome causes individuals to be exposed to an emotional exhaustion that leads to negative feelings about work and others (cynicism), with a personal sensation of ineffectiveness. This is a situation of physical, emotional and psychological exhaustion^7^ ^,^ ^33^. In this sense, it is verified that treating this syndrome with auriculotherapy is an alternative to assist in its coping.

In addition to what is herein explained, it is worth noting some reflections between the results found and the possibilities for advancements in the workers’ health area. The National Policy on Workers’ Health has, among other objectives, the opportunity to develop individual actions to recover health problems and interventions on determining factors that favor workers^48^. The diverse evidence of this systematic analysis is in line with these objectives, as it revealed that auriculotherapy is a safe practice, easy to apply, inexpensive and helps to cope with/reduce anxiety, stress and burnout. Therefore, it is urged that institutions may promote actions with this practice, for example, through sectors such as Specialized Services in Safety Engineering and Occupational Medicine (*Serviços Especializados em Engenharia de Segurança e em Medicina do Trabalho*, SESMT). 

Furthermore, the creation of a workers’ health outpatient service is another relevant and positive strategy, a place where, among other practices, auriculotherapy can be used, not only for anxiety, stress or burnout but, for example, for musculoskeletal diseases, also responsible for countless work-related ailments. In this sense, it is known that this type of service requires structural change, financial investment and specialized labor, factors that interfere with its implementation by managers. However, it is necessary to think that by promoting actions that favor workers’ health there will be a reduction in absenteeism and presenteeism, situations that will minimize institutional expenses.

Finally, it is known that systematic reviews are at the top of the levels of scientific evidence; however, the data of this analysis should be interpreted considering the methodological quality of the primary studies included. One of the review limitations consisted in the missing data from three studies, which precluded their inclusion in the meta-analysis. The high heterogeneity found in the studies limited other comparisons. A variation was identified in the way of treating (protocol) anxiety, stress and burnout with auriculotherapy. Even considering that the English language is universal, even in scientific disclosing, it should be recognized that China stands out in the use of ICPs for the treatment of the most diverse pathologies, and non-inclusion of Chinese information sources, as well as of the Mandarin language, may imply a limitation in access/selection of productions.

Although it was found that there is no standardization in the use of auriculotherapy for the treatment of anxiety, stress or burnout, a protocol is suggested, with the following: application of the practice with semi-permanent needles for eight sessions, one per week (7-day interval), in the *shen men*, brainstem, kidney, sympathetic, lung and liver points, unilaterally and with ear alternation at each session. It is recommended that this protocol be compared to a group with *sham* points and that the detection of points, for both groups, should be by means of an electrodiagnostic detector. It is also advisable to use a Chinese ear map, consider hygiene and biosafety care, and use the opposite hand to support the posterior face of the auricular pavilion. The aforementioned protocol needs to be tested and validated.

## Conclusion

It was evidenced that auriculotherapy is effective to reduce anxiety and stress in health professionals. For burnout, it is not possible to make the same assertion, given the impossibility of performing a meta-analysis; however, the studies have shown significant reductions in burnout when treated with auriculotherapy. In addition, regardless of the material used, any auriculotherapy intervention is more effective when compared to not performing it. There is diverse evidence that semi-permanent needles are more effective in reducing the outcomes evaluated, when compared to other materials or groups. Therefore, it is concluded that, when compared to control or placebo groups, auriculotherapy is effective to reduce anxiety and stress in health professionals. On the other hand, there are no studies that compare the intervention to conventional treatments. 

In this context, the evidence found in this review corroborates the literature and reasserts that auriculotherapy is an integrative practice that assists in the relief of disorders of the human organism, favoring body homeostasis. In addition, considering hospital care environments, it is verified that this practice favors workers’ health. Nevertheless, it is necessary to consider the findings since the synthesis of the evidence revealed significant heterogeneity among the studies included in the review.

Given the above, we recommend that new research studies on auriculotherapy meet the critical evaluation criteria for methodological quality. We recommend using manual structured and systematized intervention to minimize bias and, consequently, strengthen evidence for the use of this therapy in the clinical practice.

We recommend that future research studies should consider the gaps evidenced, recognizing the need for surveys evaluating the effectiveness of auriculotherapy for these three diseases simultaneously, comparing the technique to usual/conventional treatment, and using mixed-methods research. In addition, burnout needs to be explored, as the diverse evidence in relation to it was incipient.
